# Subcutaneous Injection of Mercury: “Warding Off Evil”

**DOI:** 10.1289/ehp.6891

**Published:** 2004-07-22

**Authors:** Venkat L. Prasad

**Affiliations:** Tri County Community Health Center, Dunn, North Carolina, USA

**Keywords:** case report, local abscesses, mercury injection, subcutaneous

## Abstract

Deliberate injection of mercury, especially subcutaneous injection, is rare but is seen in psychiatric patients, individuals who attempt suicide, those who are accidentally injected, and boxers who wish to build muscle bulk. Metallic mercury plays a major role in ethnic folk medicine. Neurologic and renal complications can result from high systemic levels of mercury, and subcutaneous injection usually results in sterile abscesses. Urgent surgical evacuation and close monitoring for neurologic and renal functions as well as chelation (if toxicity is indicated) are key aspects of treatment. Education of the adverse effects and dangers of mercury is important, especially in pregnant women and children. As increased immigration changes demographic patterns, proper disposal of mercury and preventing its sale and use should become urgent societal priorities. Psychiatric consultation should be obtained whenever appropriate.

## Case Presentation

Injection of elemental mercury is uncommon, and only 72 cases have been reported in the literature over the past 75 years. Of these 72 cases 46 were deliberate; most involved direct intravenous administration, usually with suicidal intent (Kayias 2003), or they were a complication of drug abuse. Bradberry et al. (1996) reported an attempted homicide by this means. Self-injection has also been reported in psychiatric patients (Soo et al. 2003), and accidental injections have been reported (Ellabban et al. 2003). Subcutaneous injection of mercury by accident (including injuries from broken thermometers), self-injection, and suicide attempts has been reported (Chodorowski et al. 1997; Ellabban et al. 2003; Smith et al. 1997; Soo et al. 2003).

A search in MEDLINE and PubMed (National Library of Medicine, Bethesda, MD) did not reveal any study or report on injection of mercury in the subcutaneous space of the hands for the sole purpose of preventing infections and “evil” during foreign travel. This practice is apparently common in several Central and South American countries. In this case report, I present such an injection received by a couple in Honduras before they traveled to the United States.

G.B., a 41-year-old Hispanic woman, and her partner, V.V., a 35-year-old Hispanic male, came to the clinic together. Both had wet towels wrapped around both their forearms and hands. They reported having pain for 5 days as well as swelling in the hands and low-grade subjective fever. The pain was localized to the dorsum of the hand and forearm, with no radiation, and was moderate in intensity and continuous, with no specific aggravating or relieving factors. The swelling and redness was localized to the same areas on the dorsum of the hand. They reported no history of bites or stings, and they had no swollen glands or joint pain. A review of systems was otherwise negative.

Both patients gave a history of having received multiple injections of mercury at a roadside nonmedical facility in Honduras about 1 week before their clinic visit. They did not know about the sterility of the procedure or if needles/syringes used were disposable. On further questioning, they indicated that the injection of mercury is a common practice among people who wish to travel abroad. The reason for their injections was to ward off “evil” and also to protect against exposure to any unknown diseases while traveling in a foreign country. The patients estimated that the injections for both hands in both patients was < US$1.00.

Both G.B. and V.V. denied any significant allergies or past medical history. They were both nonsmokers and denied alcohol or drug abuse.

A physical exam revealed G.B. to be an obese Hispanic woman in obvious distress due to pain in both hands and forearms. The general exam was unremarkable, and a local exam revealed a diffuse soft tissue swelling on the dorsum of both hands, with fluctuation, redness, and pointing (most prominent part of swelling in an abscess that marks the area of imminent rupture) in the first web space of both hands. Redness and swelling was also noted all along both forearms, with significant tenderness. No lymphadenopathy was noted. Lungs and heart were normal, and there was no renal angle tenderness and no hepatosplenomegaly. The neurologic exam was normal.

V.V. was a tall, medium-built Hispanic male in distress from pain. The general exam was unremarkable, and the local exam revealed findings similar to those for his partner, with fluctuation, redness, and tenderness in the dorsum of the hand and first web space and in the forearms. Otherwise, the exam was unremarkable.

Laboratory values for G.B. were as follows: glucose, 101 mg/dL; blood urea nitrogen (BUN), 14 mg/dL; creatinine, 0.8 mg/dL; sodium, 138 mmol/L; potassium, 4.1 mmol/L; chloride, 105 mmol/L; carbon dioxide, 22 mmol/L; calcium, 9.5 mmol/L; liver function tests, normal; white blood cell (WBC) count, 8,700/μL; hemoglobin, 12.6 g/dL; hematocrit, 37.6%; urine mercury, 11.3 μg/L; and serum mercury, < 5.0 μg/L.

Laboratory values for V.V. were as follows: glucose, 108 mg/dL; BUN, 26 mg/dL, creatinine, 1.1 mg/dL; sodium, 138 mmol/L; potassium, 4.2 mmol/L; chloride, 97 mmol/L; carbon dioxide, 26 mmol/L; calcium, 10.2 mg/dL; liver function tests, normal except for alanine aminotransferase, 64 U/L (normal, 4–60 U/L); WBC count, 8,700/μL; hemoglobin, 16.0 g/dL; hematocrit, 48.3%; and blood mercury, 100 μg/L (normal < 10 μg/L). Urine mercury analysis was not performed because V.V.’s urine samples were lost by the laboratory.

**Figure 1:**
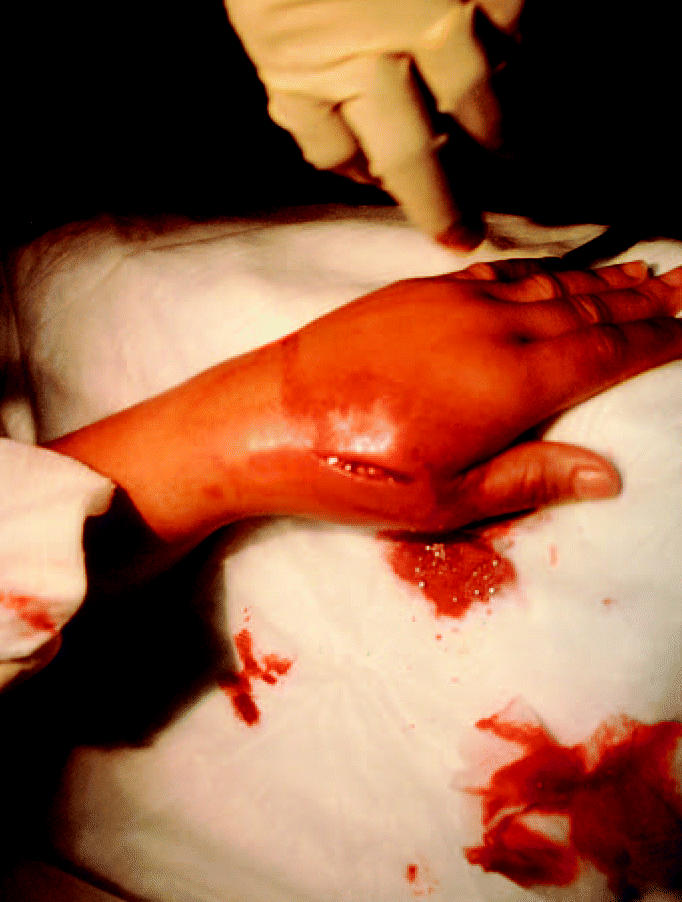
Incision made in hand of V.V. shows mercury pellets inside the incision and the inflammation of the injection site.

**Figure 2:**
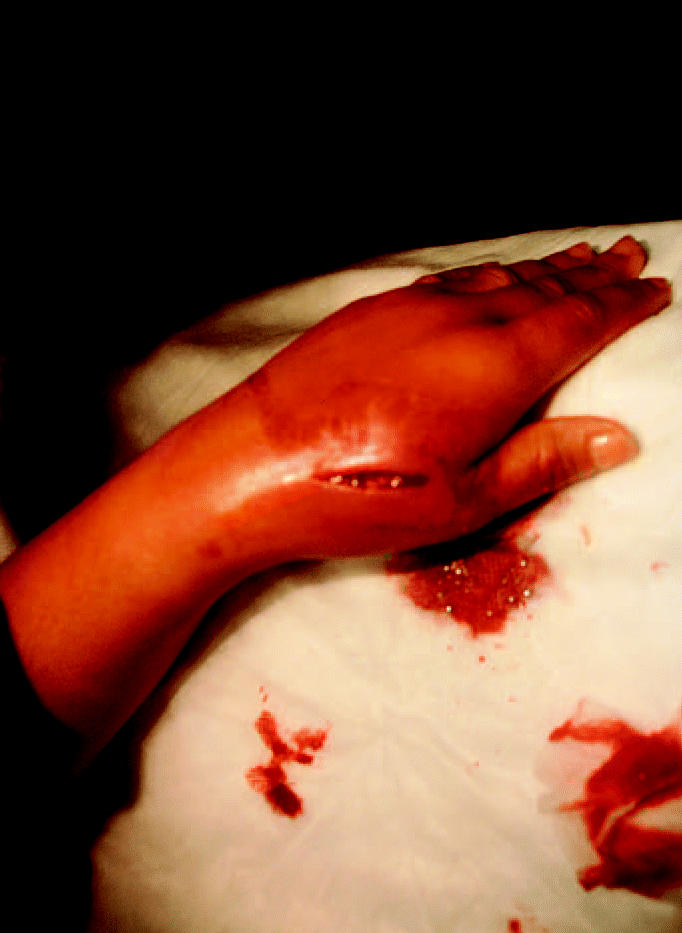
Incision site of V.V.’s hand before irrigation.

**Figure 3:**
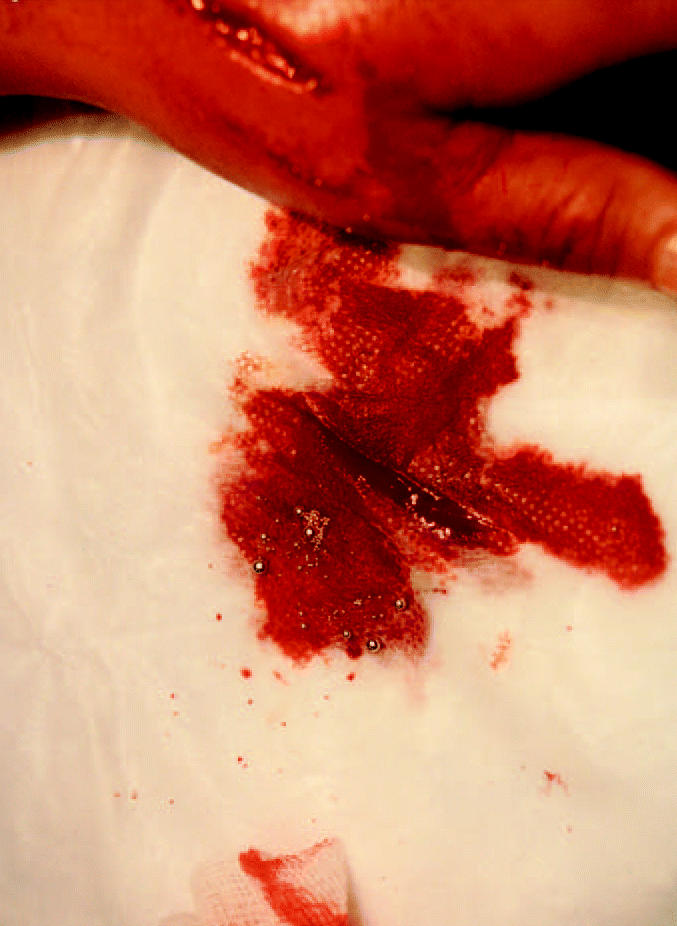
Significant amount of mercury pellets spilled during irrigation of the incision in V.V.’s hand.

A diagnosis of abscess was made, and both patients underwent incision drainage of both hands. Thick pus was evacuated along with beads of metallic mercury (Figures 1–3). Complete evacuation of all visible mercury, about 0.5 mL, was performed and wounds were thoroughly washed with copious amounts of saline. The fluid removed was sterile pus (result of milder inflammation caused by irritants, foreign bodies, etc., but not due to infection). The soaked gauze and dirty sheets were disposed in regular waste.

Postoperatively, the wounds granulated and healed well by secondary intention (left open to heal by epithelization). Since that time, the patients have been lost to follow-up.

## Discussion

Mercury is sold as “azogue” in religious stores, or botanicas, for use in Esperitismo (spiritual belief in Puerto Rico), Santeria (Cuban practices), and voodoo. The mercury is often carried personally in a pouch or spread around the house or bed, mixed in the bath, or burned in devotional candles. Mexican-Americans take it orally to relieve *empacho* (indigestion), especially in infants and children. Mercury is difficult to remove, and it can remain in carpets, walls, and homes for long periods.

The form of mercury consumed in fish is mainly methyl mercury, and mercury from occupational and dental exposure is elemental mercury. Both forms are absorbed and can have serious consequences (Magos 1997).

Concerns about mercury contamination have been growing in predominantly Hispanic and Caribbean neighborhoods. In New York City, neurotoxic levels of mercury vapor from magicoreligious and ethnomedical uses of mercury have been reported (Wendroff AP, personal communication). Wastewater samples from a residential neighborhood in Washington Heights had highly elevated mercury levels on two occasions. Secondhand exposure from previous tenants sprinkling mercury on floors also remains a problem because the contamination can remain for over a decade. Mercury exposures resulting from magicoreligious use are often greater than those occurring by eating fish or from dental amalgams (Wendroff AP, personal communication).

In a survey at the Montefiore Medical Center in New York in 1996, Zayas and Ozuah (1996) studied the sales of mercury in the Bronx area of New York City. Of the 41 botanicas they located, 38 sold elemental mercury; in 1995, 35 of the 38 botanicas sold about 25,000–155,000 capsules or vials (mean weight, 9 g) for spiritual practices. Of the users, 29.3% said that it was “sprinkled in the home” (Zayas and Ozuah 1996).

In an effort to raise the awareness among pediatricians about the possibility of toxic exposure to mercury in children, Goldman (2001) reported on the use of mercury in Santeria among immigrants from Haiti and other Caribbean nations, in which elemental mercury was sprinkled around the house. Riley et al. (2001) reported a 5% prevalence of elevated mercury levels in urine of 100 children in Bronx, New York, in August 2001. Of these children, 55% were Latino and 43% were African American (Riley et al. 2001).

In a study in Massachusetts, 898 people were surveyed in the Lawrence area, which has significant Latino and Caribbean populations (JSI Center for Environmental Health Studies 2003). The survey showed that 91 people swallowed mercury in a drink, 143 applied it to their skin, 152 burned it in candles, and 108 sprinkled it around their homes. The study authors estimated that a minimum of 6.8 lb of mercury had been released into the community through magicoreligious use. Forty percent of the Latinos in the Lawrence area knew about azogue or used it themselves. The authors were especially concerned about the large number of apartments that may have been severely contaminated.

Attempts by power companies to replace pressure-control devices for domestic gas supply has led to mercury spills, affecting 200,000 homes in one incident (Clarkson et al. 2003). High levels of mercury exposure can result from sprinkling mercury on the floor of a home or car, burning it in a candle, and mixing it with perfume. Because mercury vapor is heavy and tends to form layers close to the ground, infants and children, whose breathing zones are closest to the floor, are at highest risk. Ingested mercury passes through the gut unabsorbed. For centuries it has been used to treat constipation (Clarkson et al. 2003).

In Latin American and Central American countries, mercury is dispensed in small centers for psychic readings and in fortune telling stores, usually not a medical establishment. The entire process is very ritualistic. Clients are often requested to bathe and then have eggs smeared over their bodies. Of the various indigenous herbs and heavy metals used for treatment, mercury is popular; it is often consumed in a mixture of port wine, eggs, nutmeg, and milk. In many South American countries, mercury is often administered by intravenous injection to help athletes and boxers build muscle mass, a practice based on superstition (Smith et al. 1997).

The oral route of metallic mercury use does not cause poisoning symptoms, but its use in infants and children could cause subclinical developmental problems. Concentrations in blood and urine after ingestion of mercury remain low because very little is absorbed. However, mercury injected subcutaneously causes sterile, inflammatory, and necrotic reactions resulting in abscesses and granulomas. Environmental and occupational exposure to mercury can be determined by measuring toenail mercury levels (Garland et al. 1993; MacIntosh et al. 1997; Yoshizawa et al. 2002).

Intra-arterial injection can cause digital ischemia and/or gangrene secondary to embolization. One case of cardiac granuloma secondary to intra-arterial injection has been reported (Kedziora and Duflou 1995). When mercury is injected intravenously, it goes mainly to the lungs and can cross over to systemic circulation (Givica-Perez et al. 2001).

Cases of foreign body granuloma on the thumbs or hands have been reported after rubbing mercurial ointments (Bradberry et al. 1996). In cases of subcutaneous metallic mercury injection, patients usually present weeks to months later with an inflammatory mass at the site of injection. The diagnosis may be apparent on X-ray examination or it may be obvious at the time of surgery (Bradberry et al. 1996).

Patients may be seen remote from the mercury exposure with swelling at the injection site. Pathologic findings of granuloma, fibrosis, and histiocytes suggest a local foreign-body–type reaction to metallic mercury. Abnormal serum levels suggest that there is some lymphatic and vascular migration following subcutaneous injection (Soo et al. 2003).

Mercury can be detected by imaging X rays or ultrasound. In the case of a 32-year-old nurse who had cut the palm of her right hand with a broken thermometer 30 days earlier, sonography showed multiple small echogenic dots surrounded by a hypoechoic halo, suggesting the presence of small crystal fragments or droplets of mercury (Romero et al. 2004). No reverberation, acoustic shadowing, or flow on color or power Doppler imaging was noted. Mercury is hyperechoic on sonograms despite being liquid at room temperature. It is a safe, inexpensive, portable, and readily available imaging modality (Romero et al. 2004). Two deaths have been reported following subcutaneous injection (Chodorowski et al. 1997); cause of death was renal failure in one patient and empyema in the lung of the second patient.

There is no ban on the sale of mercury, although the Federal Hazardous Substances Act (1994) mandates that it be sold only with an attached warning label. Current U.S. public advice on disposal of mercury is confusing and inconsistent; 45% of requests for advice from local and state waste management centers resulted in advice to use regular household collections to dispose thermometers (DiCarlo et al. 2002).

Under a voluntary agreement between the U.S. Environmental Protection Agency (EPA), the American Hospital Association, Hospitals for a Healthy Environment (H2E) was formed. A pledge was made to eliminate mercury, identify pollution prevention opportunities, and reduce waste. As of March 2002, the H2E had as partners 260 hospitals, 36 clinics, 8 nursing homes, and 25 other facilities across the United States (Wendroff A, personal communication). Information on the safe disposal of mercury is available on the U.S. EPA website (U.S. EPA 2004).

With changes in demographic and population ethnic mixes, controlling the sale of mercury and ensuring its proper disposal become more urgent. Serious environmental contamination and long-term consequences could otherwise cause severe consequences in the future.
